# Correlation of Arterial CO_2_ and Respiratory Impedance Values among Subjects with COPD

**DOI:** 10.3390/jcm9092819

**Published:** 2020-08-31

**Authors:** Tomoki Uno, Tetsuya Homma, Masahiko Shigemura, Yosuke Fukuda, Tomoyuki Kimura, Chihiro Onitsuka, Tomoko Kawahara, Hiroki Sato, Kaho Akimoto, Hiromitsu Suganuma, Ayaka Kashima, Shigenori Yamamoto, Takaya Ebato, Tomohiro Matsunaga, Keisuke Kaneko, Hatsuko Mikuni, Haruna Sato, Yoshitaka Uchida, Akiko Fujiwara, Naota Kuwahara, Ryo Manabe, Hitomi Ida, Kuniaki Hirai, Yoshito Miyata, Megumi Jinno, Shin Ohta, Yasunari Kishino, Hideki Inoue, Mayumi Yamamoto, Yoshio Watanabe, Sojiro Kusumoto, Koichi Ando, Shintaro Suzuki, Akihiko Tanaka, Tohru Ohmori, Hironori Sagara

**Affiliations:** 1 Department of Medicine, Division of Respiratory Medicine and Allergology, 1-5-8 Hatanodai, Shinagawa-ku, Showa University School of Medicine, Tokyo 142-0064, Japan; unouno0926@gmail.com (T.U.); terubow0423@gmail.com (Y.F.); psofriendtomo@hotmail.com (T.K.); chisato@med.showa-u.ac.jp (C.O.); coccy.ppt.1216@gmail.com (T.K.); sato_hiro@med.showa-u.ac.jp (H.S.); kahomontresor.lien004@gmail.com (K.A.); ha.mikuni392@gmail.com (H.M.); rainbow@med.showa-u.ac.jp (H.S.); for.u.goodlife@gmail.com (Y.U.); medi123@infoseek.jp (K.H.); fourdimensions28@gmail.com (Y.M.); jinno@med.showa-u.ac.jp (M.J.); shinohta@med.showa-u.ac.jp (S.O.); ookiyookiy@med.showa-u.ac.jp (Y.K.); hiyumi2001@gmail.com (H.I.); ymym0018@med.showa-u.ac.jp (M.Y.); fzr02034@gmail.com (Y.W.); k-sojiro@med.showa-u.ac.jp (S.K.); koichi-a@med.showa-u.ac.jp (K.A.); szshintr@yahoo.co.jp (S.S.); tanakaa@med.showa-u.ac.jp (A.T.); ohmorit@med.showa-u.ac.jp (T.O.); sagarah@med.showa-u.ac.jp (H.S.); 2 Division of Pulmonary and Critical Care Medicine, Northwestern University, Chicago, IL 60611, USA; masahiko.shigemura@northwestern.edu; 3 Department of Respiratory Medicine, Asahi General Hospital, Chiba 289-2511, Japan; hiiro.gr.3@gmail.com (H.S.); guooooon@med.showa-u.ac.jp (A.K.); 4 Department of Pulmonary Medicine, Tokyo Metropolitan Health and Hospitals Corporation Ebara Hospital, Tokyo 145-0065, Japan; yamashigemotonori0715@yahoo.co.jp; 5 Department of Respiratory Medicine, Yamanashi Red Cross Hospital, Yamanashi 401-0301, Japan; doraimu2255@gmail.com; 6 Department of Medicine, Division of Allergology and Respiratory Medicine, Showa University Koto Toyosu Hospital, Tokyo 135-8577, Japan; ga_tomohiro_te@yahoo.co.jp (T.M.); kuwhrnaota@gmail.com (N.K.); manaryo1224@hotmail.co.jp (R.M.); hitomifurukawa.showauniv@gmail.com (H.I.); 7 Department of Respiratory Medicine, Ibarakihigashi National Hospital, Ibaraki 142-0064, Japan; spitz0426@gmail.com; 8 Respiratory Disease Center, Showa University Northern Yokohama Hospital, Kanagawa 224-8503, Japan; lumiere.b.324@gmail.com

**Keywords:** CO_2_, hypercapnia, FOT

## Abstract

Chronic obstructive pulmonary disease (COPD) is a respiratory illness characterized by airflow limitation and chronic respiratory symptoms with a global prevalence estimated to be more than 10% in 2010 and still on the rise. Furthermore, hypercapnic subject COPD leads to an increased risk of mortality, morbidity, and poor QoL (quality of life) than normocapnic subjects. Series of studies showed the usefulness of the forced oscillation technique (FOT) to measure small airway closure. Traditional findings suggested that hypercapnia may not be the main treating targets, but recent findings suggested that blood stream CO_2_ may lead to a worse outcome. This study aimed to seek the relationship between CO_2_ and small airway closure by using FOT. Subjects with COPD (*n* = 124; hypercapnia 22 and normocapnia 102) were analyzed for all pulmonary function values, FOT values, and arterial blood gas analysis. Student’s *t*-test, Spearman rank correlation, and multi linear regression analysis were used to analyze the data. COPD subjects with hypercapnia showed a significant increase in R5, R20, Fres, and ALX values, and a greater decrease in X5 value than normocapnic patients. Also, multiple linear regression analysis showed R5 was associated with hypercapnia. Hypercapnia may account for airway closure among subjects with COPD and this result suggests treating hypercapnia may lead to better outcomes for such a subject group.

## 1. Introduction

Chronic obstructive pulmonary disease (COPD) is a heterogenous condition with clinical presentation, mainly dyspnea, cough, sputum, and importantly, airflow limitation [1,2]. Briefly, the disease severity is defined by the degree of functional impairment of pulmonary function and (activity by) frequency of exacerbations. There is a link between disease severity and activity which forms a vicious circle that (finally) impacts patient’s quality of life (QoL) and may lead to frailty status with abnormal gas exchange, hypoxia, and hypercapnia [3,4,5].

Gaseous molecules such as oxygen (O_2_) and CO_2_ are sensed by lung cells eliciting specific responses via intracellular signaling pathways [6,7,8,9,10,11,12,13,14]. Hypercapnia, an elevation in the arterial CO_2_ tension, is generally well tolerated in mechanically ventilated patients with acute respiratory distress syndrome (ARDS), but recent studies suggest that a high concentration of CO_2_, independently of hypoxia, can act a gaso-signaling molecule resulting in adverse consequences via the alteration of transcription factor activity and microRNA (miR) expression in the lung [6,7,8,9,13,15,16,17,18,19]. Specifically, our recent study showed that high CO_2_ levels increased airway smooth muscle contractility via activation of the caspase-7-mediated miR-133a-RhoA signaling [9]. We also reported that in a small cohort of severe COPD, airway obstruction was worsened in hypercapnic patients [9]. These lines of evidence raised the possibility that elevated levels of CO_2_ within the lung (may result in an impact on) attenuated pulmonary function and lead to worse QoL in COPD [4,8,9,16].

The forced oscillation technique (FOT) is a noninvasive method and measures respiratory impedance of the airway which was first described by Dubois in 1956 [20,21,22,23,24]. FOT was able to measure and calculate the airway resistance (Rrs) and reactance (Xrs). Recent findings showed the clinical utility among asthma, cystic fibrosis, interstitial lung disease, and COPD [20,25,26,27,28,29]. Among COPD patients, previous reports showed the relationship between the early detection of acute exacerbation and physiological activity, and used this to distinguish between asthma [25,26,27,28], but the relationship between FOT and CO_2_ has not been fully investigated.

In this study, we hypothesized that elevated levels of CO_2_ could possibly influence the respiratory function values including FOT values and investigated which specific respiratory function value correlates with the level of CO_2_ levels in arterial blood. Our final aim for this study was to see the correlation between PCO_2_ and FOT values among subjects with COPD.

## 2. Methods

### 2.1. Participating COPD Subjects

Patients with chronic stable COPD with or without hypercapnia presented at Showa University Hospital meeting all the inclusion criterion: pulmonary function criteria of the global initiative for chronic obstructive lung disease guidelines, age above 40 years old, and current or ex-smoker with a smoking history of >10 pack-years. Hypercapnia was defined as PCO_2_ of more than 45 mmHg [9,30]. Clinician-based asthma diagnosed subjects were excluded. The study population, gender, age, body mass index (BMI), arterial blood gas results, and pulmonary function tests results were obtained from medical records. This study was carried out in accordance with the guidelines of the Helsinki Declaration. Study approval was granted by the ethics committee of Showa University School of Medicine (approval number, 2360) and newly enrolled patients were added to our previous study [9].

### 2.2. Arterial Blood Gas Analysis

Femoral arterial blood (1.0 mL; 3 units of heparin per syringe) was drawn with a 22-gauge needle attached to a heparinized syringe. Samples were immediately analyzed with a blood gas analyzer (Rapidlab 1200; Siemens Healthcare Diagnostics, Sudbury, United Kingdom).

### 2.3. Pulmonary Function Test and FOT

On the same examination day of arterial blood gas was taken, pulmonary function test and FOT were performed. Pulmonary function test was determined using computerized equipment (model CHESTAC-8800; Chest M.I. Co. Ltd., Tokyo, Japan) according to the recommendations of the American Thoracic Society [31]. Forced vital capacity (FVC) is the expiratory air volume after the maximum inspiration, forced expiratory volume in 1 s (FEV1) is the volume of FVC in the first 1 s, and peak expiratory flow (PEF) is the maximal flow achieved during measurement of FVC at full inspiration. Furthermore, V50 and V25 are the velocity of expiratory airflow at 50% or 25% of FVC, respectively. Finally, %FVC, %FEV1, %PEF, %V50, and %V25 stands for comparative value in (%) to normal value of each subjects.

FOT was performed with available commercial-based equipment (MostGraph-01, CHEST M.I.). The FOT procedure was performed according to major recommendations published by several authors [9,22,25,26]. On the day of the procedure, subjects were asked to wear a nose clip and were seated during tidal breathing [32]. All subjects were asked to seal their lips tightly around the mouthpiece and firmly supporting their cheeks with their hands. A minimum of three trials, each lasting 30 s, were performed. Mouth pressure and flow signals were measured and calculated for Rrs and Xrs properties against oscillatory frequency ranging from 4 to 36 Hz. We used Rrs at 5 and 20 Hz (R5 and R20, respectively), Xrs at 5 Hz (X5) for values that reflect elastic properties, resonant frequency (Fres) where Xrs crosses zero and the elastic and inertial forces are equal in magnitude and opposite, and a low-frequency reactance area (ALX), which is the integral of Xrs at 5 Hz to the Fres as previously described by other groups [22,25,26]. Briefly, R5, R20, and R5–R20 indicate the respiratory system resistance of total, central, and peripheral portions, respectively. The reactance at 5 Hz (X5) reflects the combined effect of tissue elastance and inertance. Both inspiratory and expiratory phases of oscillatory index were measured.

### 2.4. Statistical Analysis

Data are expressed as mean ± 25% of range unless specified otherwise. Values of pulmonary function test, FOT, and arterial blood gas were assessed using Student’s *t*-test, Spearman rank correlation, and multi linear regression analysis. All statistical analyses were performed using GraphPad Prism 5.0 (GraphPad Software, La Jolla, CA, USA) software.

## 3. Results

### 3.1. Characteristics of COPD Subjects

In total, 124 subjects were enrolled and analyzed (Table 1). Subjects’ median age was 76.5 years-old, female dominant with the median PCO_2_ value of 46.60 mmHg in the group of subjects with hypercapnic COPD. Whereas, median age of 73 years-old, male dominant, and median PCO_2_ value of 36.55 mmHg in the subjects with normocapnic COPD group. Arterial blood gas values were distinct for each other group, whereas background characteristics were not significantly different.

### 3.2. Pulmonary Function Test and FOT Values of Enrolled Subjects with COPD

Most of the pulmonary function test values were significantly lower in the subjects with hypercapnic COPD group than normocapnic COPD group; FVC, %FVC, FEV1, %FEV1, %PEF, V25, and %V25 (Table 2). Only R5 and R5-20 values showed a consistent difference in all phases (whole breath, inspiratory, and expiratory) when both groups were compared (Table 3). The whole breath of R5, R5–R20, Fres, and ALX values showed higher subjects with hypercapnia. Meanwhile, X5 value showed higher in subjects with non-hypercapnia.

### 3.3. Correlation of PCO_2_ and Pulmonary Function Test and FOT Values

**Figure 1:**
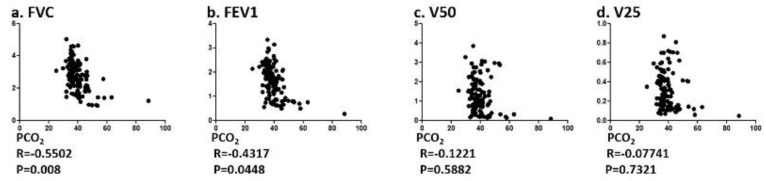
Correlation between PCO_2_ and pulmonary function test values. Specifically, correlation between PCO_2_ and (**a**) FVC, (**b**) FEV1, (**c**) V50, and (**d**) V25 were analyzed using Spearman rank correlation test.

**Figure 2:**
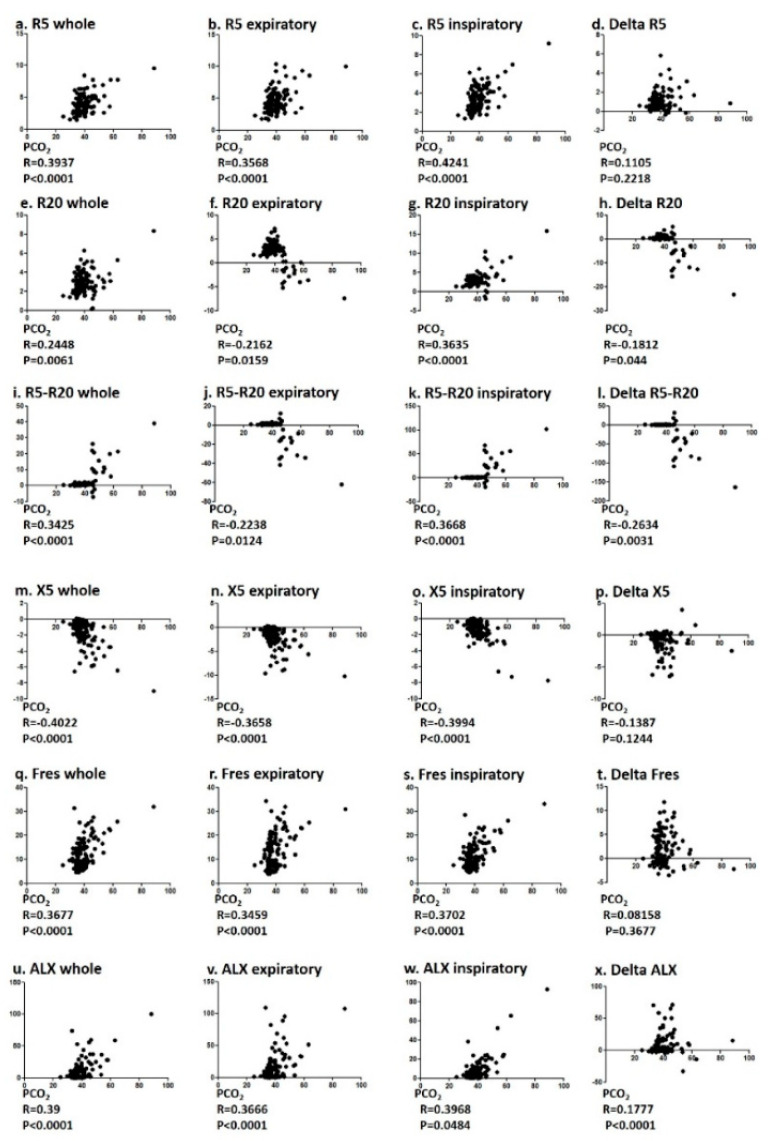
Correlation between PCO_2_ and FOT values. Specifically, correlation between PCO_2_ (**a**–**d**) R5, (**e**–**h**) R20, (**i**–**l**) R5-R20, (**m**–**p**) X5, (**q**–**t**) Fres, and (**u**–**x**) ALX were analyzed using Spearman rank correlation test.

The correlation between PCO_2_ and pulmonary function values (FVC and FEV1) were correlated with significance, but both V50 and V25 did not (Figure 1). R5, R20, R5–R20, X5, Fres, and the ALX in whole breath phase showed a significant correlation with PCO_2_ (Figure 2). Among FOT values, R5 in the inspiratory phase and X5 in the whole breath phase showed a significant correlation with relatively strong R values of 0.4241 and −0.422, respectively.

### 3.4. Multiple Linear Regression Analysis of Clinical Variables Associated with Hypercapnia

Multiple linear regression analysis of hypercapnia-positivity was performed using eight variables (Table 4). The hypercapnia-positivity was independently predicted by the whole breath of R5 values.

## 4. Discussion

In this current study, we assessed the correlation between each FOT values and PCO_2_ among COPD subjects. We found that FOT values largely differ between COPD subjects with or without hypercapnia. Although the values of pulmonary function test also correlated significantly with PCO_2_, the correlation was also significant using the values of FOT, which were easier to obtain than conventional pulmonary function tests.

Current COPD guideline diagnosis process relies on using spirometry to assess airflow limitation with symptoms and exacerbation history [33]. Spirometry involves a forced expiratory operation to measure each value such as FEV1 [24]. This procedure cannot be found in real life and often gives stress to low and high-aged population. It is true that the forced expiratory flow between 25 and 75% of FVC is one of the represented values of small airway closure, but the repeated process can make a large difference. To solve these problems, FOT was developed in subjects with asthma and COPD, and for pediatrics subjects [34]. The current study showed the correlation of arterial CO_2_ and small airway closure, suggesting measuring FOT maybe useful for monitoring arterial CO_2_, since obtaining arterial blood gas sample is time taking and invasive in some degree. Further studies will be needed to assess the usefulness of FOT in subjects with hypercapnia.

The correlation between respiratory impedance and pulmonary function has been reported [35,36,37,38,39]. Lower FEV1 /FVC and altered peripheral FOT measures (X5 and Fres) were associated with uncontrolled asthma [36]. Previous studies showed that correlations between Rrs and Xrs values and FEV1 and FEV1/FVC, while Matsumoto et al. found the correlations between alveolar NO levels and R5–R20, X5, and ALX [37,38]. Williamson et al. also found weak correlations between fractions of exhaled NO and FOT values, R5–R20, and Fres in patients with asthma or COPD [39]. Additionally, in a cohort of 215 patients GOLD stages 1 to 4, values for ALX was related to the ratio of residual volume to total lung capacity (RV/TLC), leading to inferring the degree of air trapping is related to reduced lung compliance [24,40]. Other studies demonstrated the relationship between increased value of ALX and the exacerbation rate of COPD [40,41]. Multiple regression analysis of 75 patients with moderate COPD found that R5–R20 was more related to health status and symptoms than either FEV1 or HRCT low attenuation [42]. Even in the early stage of subjects with COPD, among 124 subjects who had positive spirometry criteria, the presence of self-reported symptoms was associated with higher values of R5–R20 and ALX [43]. Our current study showed positive correlation between PCO_2_ and R5, R20, R5–R20, Fres, and ALX. X5 was the only factor that negatively correlated and showed a relatively high value of correlation. X5 is thought to reflect the combined effect of tissue elastance and inertance, therefore not only caused of COPD, mainly tobacco smoke, but CO_2_ may affect the tissue remodeling. Since there was correlation between PCO_2_ and conventional pulmonary function test or FOT from our current study, it is fair to say that airway closure is evident in some degree. Also, our previous findings showed that this action is partly due to caspase-7-mediated miR-133a-RhoA signaling [6,9].

Our study showed the degree of significant hypoxia was greater in subjects with hypercapnia as compared to normoxia than without hypercapnia. Two trials undertaken in the 1970s showed a decrease in mortality in patients with COPD and severe resting hypoxemia when receiving long-term oxygen therapy (LTOT) compared to nocturnal oxygen therapy or control [44,45]. In a more recent study from the Long-Term Oxygen Treatment Trial Research Group, patients with stable COPD and resting or exercise-induced moderate desaturation had no benefit of LTOT versus the no supplemental oxygen group in terms of mortality, exacerbations or functional status, suggesting PO_2_ level is not the only factor that regulate those outcomes in severe COPD [46]. The widely discussed study by Köhnlein et al. showed a dramatic improvement of 1-year mortality (12% versus 33%) in patients with hypercapnic, stable COPD when home mechanical ventilation was targeted to reduce hypercapnia, suggesting the importance of PCO_2_ level in treating COPD patients [47]. A recent report by Suh et al. investigated whether FOT-based auto-titration of end-expiratory pressure (EPAP) would abolish or minimize tidal-breathing expiratory flow limitation among subjects with COPD and chronic respiratory failure [48]. Although participated patients were small, what they found was that FOT-based algorithm abolished tidal-breathing expiratory flow limitation followed by minimizing transdiaphragmatic pressure swing and neural respiratory drive (NRD) in the study. It was also reported that NRD is related to PCO_2_ and removal of PCO_2_ may further reduce the mortality rate in the acute settings [17]. From our own and previous studies, an FOT-based and decreasing PCO_2_ approach may improve parameters of both acute and chronic settings.

**Figure 3:**
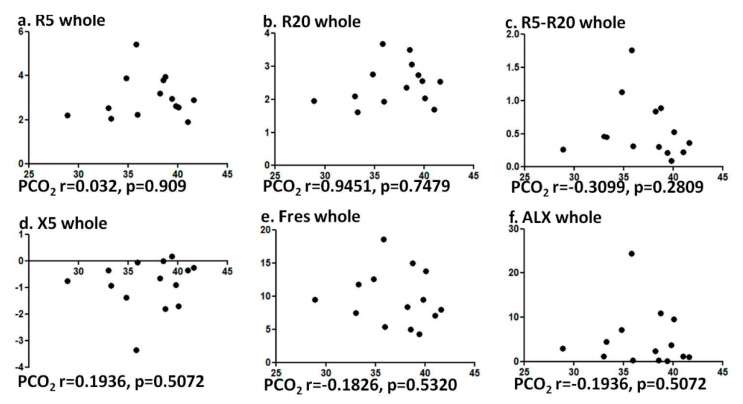
Correlation was not observed between PCO_2_ and FOT values among healthy patients. Specifically, correlation between PCO_2_ and (**a**) R5, (**b**) R20, (**c**) R5-R20, (**d**) X5, (**e**) Fres, and (**f**) ALX were analyzed using Spearman rank correlation test.

The current study has some limitations. The number of subjects enrolled in the study was not large, but large enough when compared to past studies. Although subjects’ current medication was not accounted for this study, there is not much of the study pointing out that inhaled agents, such as inhaled corticosteroids, long-acting beta-agonists, and long-acting anticholinergics, could alter the gas exchange. We cannot deny the possibility that hypoxia may affect the pulmonary function values in our subjects. Our prior study showed the role of elevated CO_2_ for airway contractility, independently of hypoxia [9]. This study showed that the relation between PCO_2_ level and FOT values in healthy volunteers was not evident (Figure 3), suggesting that changes in CO_2_ levels at normal PCO_2_ range may not affect pulmonary functions.

The term ‘permissive hypercapnia’ has been proposed for a while and the strategy is currently accepted in treating patients [18,19,49]. It has been increasingly evident that elevated CO_2_ acts as a signaling molecule with unwanted effects on the local lung and whole body [15,47,49,50,51,52,53,54]. Further preclinical (basic) and clinical studies are warranted to define who should benefit from treating or leaving hypercapnia (for subjects with acute to chronic) in lung diseases. Since one of the targets to treat subjects with COPD is to evaluate the narrowed small airways, the current study supports targeting to correct PCO_2_ level as the new strategy [48] to treat subjects with COPD and hypercapnia.

## Figures and Tables

**Table 1:** Background characteristics of enrolled subjects with COPD.

	Hypercapnia	Normocapnia	*p* Value
	(*n* = 22)	(*n* = 102)	
Age, years-old	76.5 (68.25–80.75)	73 (67.25–77.75)	0.245
Gender, male/female, n	9/13	74/28	0.303
Smoking history, pack-years	55 (32.5–105.12)	54 (39–91.87)	0.816
Hight, cm	157.35 (151.72–168)	161 (154.37–167.22)	0.786
Body weight, kg	52.85 (48.17–67.25)	57.9 (50.15–68.8)	0.252
BMI, kg/m^2^	20.88 (18.66–25.60)	23.36 (20.11–25.74)	0.18
Body surface area, m^2^/kg^2^	1.53 (1.45–1.69)	1.58 (1.47–1.75)	0.308
Arterial blood gas			
pH	7.42 (7.39–7.42)	7.44 (7.43–7.46)	<0.001
pCO_2_, mmHg	46.60 (45.7–53.2)	36.55 (35–39.7)	<0.001
pO_2_, mmHg	71.2 (67.8–84.52)	84.55 (75.62–90.45)	<0.003
HCO_3_^-^, mmol/L	30 (27.97–32.52)	24.9 (23.32–26.37)	<0.001
BE, mmol/L	4.1 (2.95–7.5)	0.9 (−0.3–2.2)	<0.001
tCO_2_, mmHg	26 (24–27.7)	30.75 (29.2–33.82)	<0.001

**Table 2:** Pulmonary function test values of enrolled subjects with COPD.

	Hypercapnia	Normocapnia	*p* Value
	(*n* = 22)	(*n* = 102)	
FVC, L	1.97 (1.40–2.57)	2.89 (2.33–3.47)	<0.001
%FVC, %	65.85 (51.40–82)	97.3 (84.85–110.07)	<0.001
FEV1, L	0.8 (0.66–1.31)	1.73 (1.32–2.19)	<0.001
%FEV1, %	36.82 (29.1–62.95)	74.7 (55.12–84.45)	<0.001
FEV1%, %	53.71 (31.88–68.94)	63.15 (50.84–71.19)	0.228
PEF, L	2.98 (2.54–4.18)	5.11 (3.76–6.69)	0.781
%PEF, %	39.20 (29.55–39.20)	69.35 (48.02–81.15)	<0.001
V50, L	0.58 (0.21–1.43)	1.15 (0.65–1.89)	0.044
%V50, %	18.35 (7.15–67.75)	35.05 (19.27–61.4)	0.101
V25, L	0.14 (0.12–0.34)	0.26 (0.16–0.42)	0.006
%V25, %	18.1 (10.72–29.27)	27.2 (16.22–39.57)	0.015

**Table 3:** FOT values of enrolled subjects with COPD.

	Hypercapnia	Normocapnia	*p* Value
	(*n* = 22)	(*n* = 102)	
R5, cmH_2_O/L/s			
Whole breath	5.05 (3.58–6.43)	3.50 (2.72–4.32)	0.001
Inspiratory	5.56 (3.62–7.75)	3.91 (3.00–5.02)	0.004
Exspiratory	4.32 (3.28–5.07)	3.09 (2.34–3.83)	0.005
delta R5	1.17 (0.25–2.07)	0.77 (0.47–1.29)	0.001
R20, cmH_2_O/L/s			
Whole breath	2.78 (2.01–3.77)	2.56 (2.21–3.31)	0.891
Inspiratory	−1.47 (−3.45–0.22)	2.87 (2.33–3.62)	<0.001
Exspiratory	3.94 (1.87–7.51)	2.34 (1.90–3.06)	0.009
delta R20	−5.46 (−11.25–−0.21)	0.41 (0.22–0.75)	<0.001
R5–R20, cmH_2_O/L/s			
Whole breath	9.29 (2.54–18.76)	0.80 (0.53–1.19)	<0.001
Inspiratory	−14.75 (−30.01–−1.01)	1.00 (0.66–1.47)	<0.001
Exspiratory	24.04 (3.71–48.77)	0.57 (0.34–0.89)	<0.001
delta (R5-R20)	−38.78 (−78.77–−2.98)	0.38 (0.18–0.58)	<0.001
X5, cmH_2_O/L/s			
Whole breath	−2.66 (−4.51–−1.12)	−0.74 (−1.90–−0.37)	<0.001
Inspiratory	−3.34 (−5.26–−1.09)	−0.78 (−2.41–−0.35)	<0.001
Exspiratory	−1.96 (−3.01–−1.16)	−0.74 (−1.34–−0.39)	<0.001
delta X5	−0.79 (−2.29–0.09)	−0.09 (−0.96–0.18)	0.108
Fres, Hz			
Whole breath	19.03 (11.99–22.57)	9.90 (7.44–15.05)	<0.001
Inspiratory	20.87 (12.16–23.89)	10.47 (7.46–16.73)	<0.001
Exspiratory	16.76 (11.66–22.18)	9.81 (7.34–13.04)	<0.001
delta Fres	2.34 (0.89–4.35)	1.18 (−0.62–4.14)	0.431
ALX, cmH_2_O/L/s Hz			
Whole breath	22.70 (5.17–36.94)	2.91 (1.24–12.16)	<0.001
Inspiratory	29.53 (5.20–47.61)	2.98 (1.12–16.00)	<0.001
Exspiratory	14.31 (5.75–23.49)	2.92 (1.28–7.25)	<0.001
delta ALX	7.76 (0.81–18.79)	0.74 (−0.47–7.24)	0.059

**Table 4:** Multiple linear regression analysis of clinical variables associated with hypercapnia.

	Coefficient	SE	95%CI	*p* Value
Age	−0.101	0.064	−0.229 to 0.026	0.118
Gender	2.127	1.292	−0.431 to 4.687	0.102
BMI	−0.214	0.269	−0.474 to 0.046	0.105
R5 whole breath	1.46	0.679	0.114 to 2.805	0.033
R20 whole breath	−1.138	0.693	−2.5116 to 0.234	0.103
Fres whole breath	−0.414	0.269	−0.948 to 0.118	0.126
X5 whole breath	−1.482	2.231	−5.903 to 2.938	0.507
ALX whole breath	0.21	0.179	−0.145 to 0.566	0.244
